# Prenatal Genetic Testing in the Era of Next Generation Sequencing: A One-Center Canadian Experience

**DOI:** 10.3390/genes13112019

**Published:** 2022-11-03

**Authors:** Asra Almubarak, Dan Zhang, Mackenzie Kosak, Sarah Rathwell, Jasmine Doonanco, Alison J. Eaton, Peter Kannu, Joanna Lazier, Monique Lui, Karen Y. Niederhoffer, Melissa J. MacPherson, Melissa Sorsdahl, Oana Caluseriu

**Affiliations:** 1 Department of Medical Genetics, University of Alberta, Edmonton, AB T6G 2H7, Canada; 2 Women’s and Children’s Health Research Institute, University of Alberta, Edmonton, AB T6G 1C9, Canada; 3 Maternal Fetal Medicine Clinic, Royal Alexandra Hospital, Edmonton, AB T5H 3V9, Canada; 4 Regional Genetics Program, Children’s Hospital of Eastern Ontario, Ottawa, ON K1H 8L1, Canada

**Keywords:** prenatal, genetic testing, postnatal, chromosomal microarray, gene panel, whole exome sequencing, next generation sequencing, pregnancy

## Abstract

The introduction of next generation sequencing (NGS) technologies has revolutionized the practice of Medical Genetics, and despite initial reticence in its application to prenatal genetics (PG), it is becoming gradually routine, subject to availability. Guidance for the clinical implementation of NGS in PG, in particular whole exome sequencing (ES), has been provided by several professional societies with multiple clinical studies quoting a wide range of testing yields. ES was introduced in our tertiary care center in 2017; however, its use in relation to prenatally assessed cases has been limited to the postnatal period. In this study, we review our approach to prenatal testing including the use of microarray (CMA), and NGS technology (gene panels, ES) over a period of three years. The overall diagnostic yield was 30.4%, with 43.2% of those diagnoses being obtained through CMA, and the majority by using NGS technology (42% through gene panels and 16.6% by ES testing, respectively). Of these, 43.4% of the diagnoses were obtained during ongoing pregnancies. Seventy percent of the abnormal pregnancies tested went undiagnosed. We are providing a contemporary, one tertiary care center retrospective view of a real-life PG practice in the context of an evolving use of NGS within a Canadian public health care system that may apply to many similar jurisdictions around the world.

## 1. Introduction

Congenital anomalies impact approximately 2–4% of pregnancies and lead to structural or functional abnormalities [1], with severe consequences including intrauterine and newborn demise, chronic diseases and lifelong disability, posing a significant burden on the families affected and the medical system [2]. Modalities of prenatal testing that lead to diagnoses and facilitate decision making have evolved significantly over the past decade as part of the unprecedented progress registered in the field of genetics. The armamentarium in prenatal genetics (PG) includes both targeted tests (QF-PCR, gene panels) and genomic approaches at different levels of resolution (karyotype, chromosomal microarray, whole exome and whole genome sequencing). The tests used are based on internal clinical protocols (first line testing is for the common aneuploidies by screening and then diagnostic tests), but also based on fetal abnormalities identified by various imaging methods (ultrasound, echocardiogram, MRI), family history [3] and parental choices [4] and in keeping with medical systems availability. Up to 30% of diagnoses are achieved by karyotype [5], and an additional 4–6% by chromosomal microarray [6]. That leaves more than half of the fetal cases with anomalies undiagnosed [7]. The gradual introduction of next generation sequencing (NGS) over the past decade has marked a shift in the approach to genetic diagnosis either by a targeted gene panel approach that incorporates a differential diagnosis when a diagnostic handle can be identified, or by a comprehensive genomic approach when the diagnosis is non-specific or too complex through exome (ES) and, exceedingly rare at the moment, genome sequencing.

Exome sequencing does not require prior knowledge regarding the potential causative gene for an observed phenotype [4]. Therefore, it appears to be particularly well poised in a prenatal context. Its use is generally accepted as a powerful tool in pediatric [8] and adult genetics [9], and has slowly expanded to the area of prenatal diagnosis. Its initial reluctance being explained by unknown or incompletely understood prenatal phenotypes in relation to interpretation of DNA variants [10]. Guidance for the clinical implementation of NGS in PG is supported by several professional associations [4,11,12]. Over the past few years, a flurry of studies has showed a wide range of diagnostic yields anywhere between 6% [13] and 89% [14], demonstrating its overall clinical utility [15], with a pooled yield of 31% as showed by a recent meta-analysis [16]. Discrepancies in achieving a diagnosis by ES between studies are reflected in the variable inclusion criteria, study cohort size, and interpretation of results, amongst other factors [16]. Despite all supportive evidence, the reality of prenatal diagnostic practice is that its application remains inconsistent, including countries with publicly available medical systems as is the case in Canada. We have evaluated the prenatal diagnostic yields in our tertiary care center over a period of three years, since ES has been clinically available postnatally, and are describing the range of diagnoses achieved with a focus on those obtained using NGS.

## 2. Materials and Methods

Alberta is a province in Western Canada with a population of approximately 4 million individuals and about 50,000 births a year [17]. Much of this population is centered in two major urban communities, in Calgary and Edmonton [17]. Our center, Edmonton, receives referrals from the Northern half of the province, including Metro Edmonton area (1.1 million), and neighboring provinces of Western Saskatchewan, Eastern British Columbia, Nunavut and Northwest Territories serving a total population of over 2 million people [17]. All permanent residents or Canadian citizens have access to a public health care system, and the modalities of genetic testing reviewed by this study are covered as part of publicly funded medical care [18].

We have performed a retrospective chart review of 450 consecutive cases referred and assessed during the pregnancy by geneticist evaluation between May 2017 and June 2020. The service of the geneticists was utilized on a case-by-case basis and requested by the Maternal Fetal Medicine Clinic at the Royal Alexandra Hospital in Edmonton, AB. Genetic counsellors work with MFM specialists who order first trimester screens and follow up diagnostic tests especially in the first trimester. Rapid aneuploidy detection is completed either by CVS or amniocentesis; in cases with anomalies that have normal results, or if the findings are not expected to be explained by a common aneuploidy, referral to a geneticist for further evaluation is made. Our study included a review of the modalities of genetic testing ordered by geneticists including chromosomal microarray (CMA) utilized prenatally in our center since 2015, and next generation sequencing (NGS)-based tests. If a diagnosis was not achieved through CMA or the CMA results did not explain the fetal phenotype, further testing was ordered by gene panel testing. Since May 2017, ES has become a clinically available test in our center for postnatal cases. This allowed us to use ES in fetuses including stillbirths, terminations of pregnancy, fetal demises, and newborns. The chart reviews included information regarding reason for referral, maternal age and gestational age at referral, maternal and family history, fetal and postnatal findings by imaging modalities (ultrasound, fetal echocardiogram, MRI), type of tests completed including CMA, gene panels, ES, outcomes of the pregnancy, and management implications. While all the cases reviewed were assessed during an ongoing pregnancy, tests were completed at different stages either prenatally (CMA, panels) or postnatally (CMA, panels, ES). The intricacies of real-life practice in PG, reflected in the way genetic testing has been completed following a prenatal consultation, are captured in Supplementary data, Figures S3–S5. For the results presented, variants of unknown significance (VUS) were included together with the negative results as no management decisions could be made at the time they were obtained. Data was collected in a Microsoft Excel spreadsheet and analyzed using SAS Ver9.4. All variables were reported as median (IQR) for continuous variables, and percentages (%) for categorical variables.

## 3. Results

**Figure 1:**
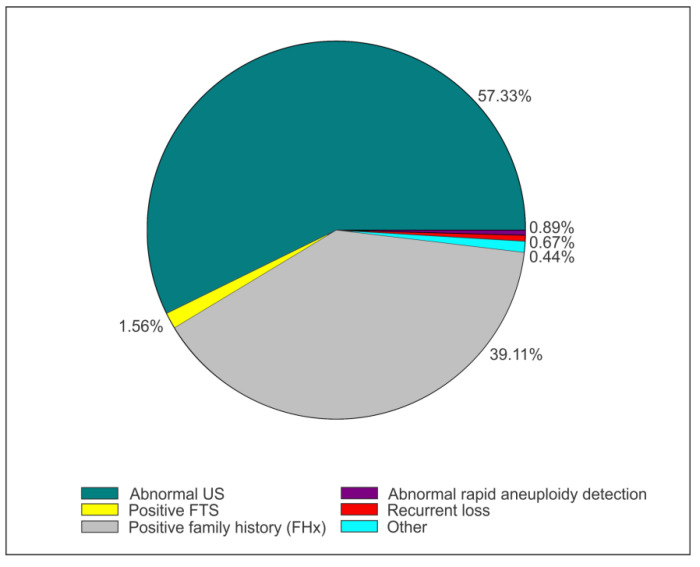
A pie chart representing the proportion of reasons for referral to our clinic between May 2017 and June 2020 with fetal structural abnormalities (57.33%) and positive family history (39.11%) as the common reasons, followed by positive first trimester screen (1.56%), abnormal rapid aneuploidy detection (0.89%), Recurrent Loss (0.67%) and other reasons (0.44%).

Four hundred and fifty patient charts were reviewed for the mentioned timeline. The majority of cases 258 (57.3%) were referred for fetal abnormalities, followed by 176 (39.11%) of cases referred for pertinent family history that incorporates maternal and paternal positive history, previous child (PC) or previous pregnancy (PP) with abnormal findings. Other reasons for referral included a positive first trimester screen (FTS), an abnormal result on Rapid Aneuploidy Detection, or a recurrent loss (Figure 1).

The average maternal age of cases reviewed was 31 (27–35) years old, and prenatal testing was done at a median gestational age (GA) of 20 weeks. Aside from abnormal ultrasound findings, a total of 136 pregnant women received fetal echocardiography, of which 52 (38.2%; 52/136) were abnormal. Fetal magnetic resonance imaging (MRI) was completed on 51 fetuses and abnormalities were identified in 41 (80.4%; 41/51).

A total of 408 fetal records were reviewed. The majority of fetal records were positive for at least one anomaly involving amniotic fluid, head & neck, central nervous system (CNS), thorax, cardiovascular (CV), gastrointestinal (GI), genitourinary, and musculoskeletal (MSK) systems. Moreover, fetal records indicated that 290 (71.1%) cases had a single organ system involved, whereas 118 (28.9%) of the results showed the involvement of multiple systems.

294 (72.6%) of fetuses were carried through successful pregnancies, while 63 (15.6%) of pregnancies were terminated, 33 (8.1%) resulted in stillbirths, and seven (1.7%) resulted in neonatal deaths.

**Figure 2:**
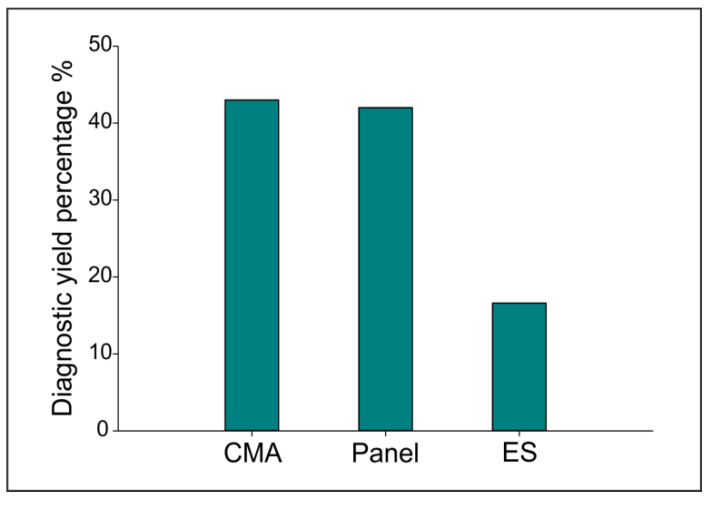
CMA and gene panel testing has the highest diagnostic yield in our cohort. A bar graph of CMA, gene panel and ES diagnostic yield percentages in our cohort.

We were able to complete testing in a total of 250 patients in our cohort, achieving a diagnosis in 73 patients (for a total of 76 positive results, with three patients having a double diagnosis, see below for details) with an overall yield of diagnosis of 30.4% (76/250). Within the diagnoses obtained, 43.2% were represented by CMA testing (33/76), 42% were obtained through a panel (32/76), and 16.6% by ES (11/76; Figure 2) for a total yield of 56.6% by NGS technologies (43/76). Of these, 43.4% (33/76) of the diagnoses were obtained during ongoing pregnancies while the majorities were tested in the postpartum period (Figure S1).

For all the CMA performed (225), the overall positive results were obtained in 33 cases for a yield of the test of 14.6% (33/225). Of all the CMA performed 70.6% (159/225) were completed during the prenatal time with a positive diagnostic yield of 12.5% (20/159). Postnatally, a further 66 CMA were completed with a positive diagnosis in 19.6% (13/66) (Figures S3–S5).

For all the panels performed (92), the overall diagnostic yield was 34.7% (32/92) (see Table 1). Forty-seven point eight of these tests (44) were completed during ongoing pregnancies with a positive diagnosis in 29.5% (13), while the postnatally completed panel testing (48) reached a diagnosis in 39.5% (19) of the cases. Most cases (5/13; 38.4%) diagnosed prenatally were represented by skeletal dysplasias (SD), followed by Noonan syndrome (3/13; 33%), and five other diagnoses involved an overgrowth syndrome, skin disorder, one immunodeficiency, one neurological disorder, and one GI disorder (see Table 1 for details). The same trend was noticed on positive panel results obtained postnatally, with 42% of the cases being SD diagnoses, followed by 21% (4/190) cases of craniofacial disorders, and 15.7% (3/190) being represented by neurological conditions and other single diagnoses (see Table 1 for details). The decision to order a panel was based on ultrasound findings in the majority of cases diagnosed prenatally (84.6%) vs. postnatally (52.6%), and a positive family history for the remainder of cases in both settings respectively. Out of the panel diagnoses achieved prenatally, 38.4% are in living children, 23% underwent termination of pregnancy (TOP), 23% were stillbirths, and one case was a neonatal demise (7.6%). For postnatally diagnosed cases by panel, the majority, 57.9% are living, 21% were diagnosed following a TOP, and 21% were neonatal demises.

**Figure 3:**
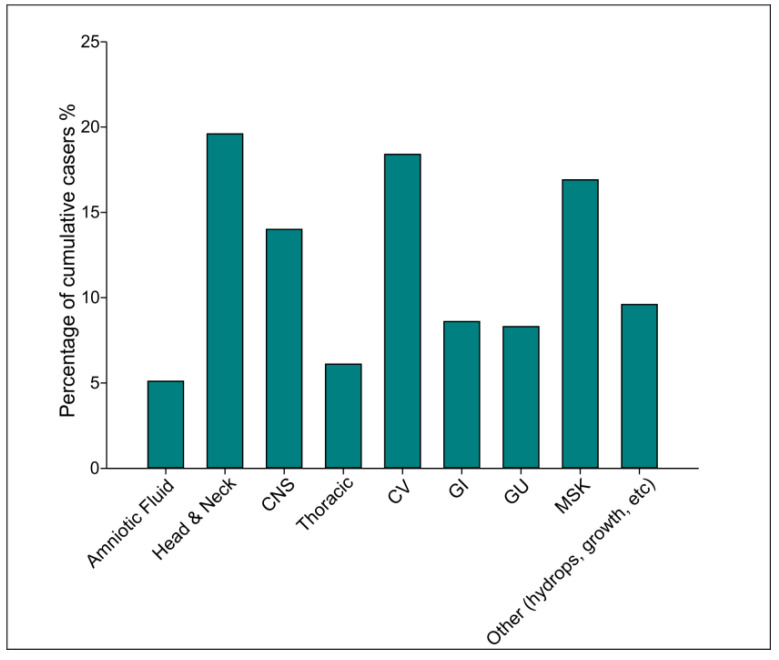
Cumulative distribution of fetal features in cases with positive NGS-based test results. A bar graph of the distribution of fetal features of cumulative cases who had prenatal and postnatal panel, and postnatal ES tests.

ES was completed only after the end of the pregnancy in a total of 38 cases, with a positive rate of 28.9% (11/38; Table 2) in nine patients (23.6%), and four (10.5%) had uncertain results. Three of the ES positive cases had a double diagnosis (see Section 4), the second diagnosis being achieved by ES in two cases and by CMA in one case. In all the cases, decision to perform the test was based primarily on abnormal ultrasound findings, followed by completion of a combination of negative tests (RAD, CMA, +/-panel). Almost half of the diagnoses obtained (44%) involved one system based on prenatal information, with all the positive cases being multisystemic on a further postnatal evaluation. Out of the patients with a positive diagnosis achieved through ES, more than half (55.5%; 5/90) are living, two pregnancies were tested following TOP, and two of the newborns underwent demise early postnatally (Table 2). A distribution of fetal features in cases with positive tests are illustrated in Figure 3 and Supplementary Figure S2.

## 4. Discussion

This retrospective chart review was performed in a Canadian publicly funded tertiary care center that has gained access to different means of prenatal diagnostic tools over time (CMA in 2015; postnatal ES in 2017). Our study evaluates and compares the diagnostic yields of three different genetic tests: CMA, and NGS-based gene panels and ES. Our review has included the referrals received for geneticist assessment during prenatal time while testing has been asynchronous (Supplementary Figures S3–S5). Thus, this study provides a transparent look at a real-world clinical PG practice scenario and how diagnoses were achieved in a clinical setting (Supplementary Figures S3–S5). The literature tendency is to focus on individual testing modalities as they become available in research or clinical settings, being either CMA or ES, or for specific phenotypes with gene panel sequencing use, but there is little literature in a more global overview of testing modalities in real-life settings.

Testing was performed in slightly more than half of the cases evaluated in our cohort (55.5%). While the majority of the tests were completed prenatally (203 vs. 152) the higher yield was achieved postnatally (41 of the positive tests vs. 35 positive tests prenatally). This may be explained by the fact that CMA has been both recommended as a first tier diagnostic tool in the evaluation of fetal structural anomalies [19] and has been routinely performed in our clinic for a longer time compared to other tests. Diagnoses achieved by CMA was higher (14.6%) than what reported in the literature, possibly due to a case selection bias favoring referrals for pregnancies with fetal imaging anomalies, as this has been consistently indicated in our cohort overall and in the proportion of cases tested with positive results. Our cohort is comprised of women with a median age below 36 and expected to have more copy number variants (CNV) than aneuploidies [20].

The highest yield of diagnoses by test type has been obtained by using panels and this reflects the nature of the test, utilized in cases with a good diagnostic handle and represented in our study disproportionately by cases with SD, confirming a previously recognized high rate of pick up by NGS in PG [14]. Our findings underline the need of maintaining panel sequencing as a tool in PG when there is a well-defined phenotype or positive family history that helps in sufficiently narrowing the differential diagnosis. This test modality limits the number of VUSs and costs less than ES which is an important consideration in a publically funded health care system. We show that our approach in utilizing ES postnatally, has led to an overall yield of 28.9%, lower than expected based on the latest evidence in the literature [16], and enhanced by the two double ES diagnoses. The highest overall diagnostic yield in our cohort was obtained by NGS technologies (gene panels and ES), the majority of the diagnostic results were obtained postnatally. That is only in part explained by the fact that WES was available postnatally, as the majority of gene panels, available in ongoing pregnancies, were also completed postnatally, in born children. In fact, while the majority of the CMA tests were completed prenatally, only half of the gene panels were done in ongoing pregnancies. It will be important to observe over time, as the public becomes familiarized with more diagnostic tools would the prenatal testing increase? Our practice is impacted by the challenging follow up with families in cases of demise and TOP that precluded some couples initially willing to stay involved to continue the diagnostic odyssey. Although our cohort was small, we were surprised to find three cases with a double diagnosis.

Case 67893001 (see Table 2) was an out of province family, and the only one that had ES during pregnancy as per the home province PG practice. The family decided to have their care continued in our center, for a fetal finding of absent ductus venosus, persistent right umbilical vein, with portosystemic shunting into the right iliac vein. The fetus was found to carry a pathogenic *RASA1* variant, shared with his mother who did not have any abnormalities identified despite extensive imaging, and a de novo VUS in *KMT2C* gene. Postnatal evaluation showed relative macrocephaly and distinct facial features (prominent forehead, apparent hypertelorism, bilateral epicanthal folds, horizontal palpebral fissures, short nose, hypoplastic bridge, bulbous tip, anteverted nares; open mouth, Cupid bow upper lip), neonatal hypotonia, and failed hearing test. Moderate bilateral sensorineural hearing loss was confirmed and hearing aids implemented. During a zoom follow up at the age of 3 years, distinctive facial features were noticed again as well as mild gross and fine motor delays, as well as moderate speech delay. A diagnosis of Kleefstra syndrome type 2 is clinically entertained and functional studies are underway to clarify the nature of the initial VUS.

The mother of Case 67295001 was seen for a family history of spinal muscular atrophy (SMA) at 36 weeks gestation with reduced fetal movements and breech presentation. No genetic testing was completed previously. Testing was discussed but the patient went into labour before the clinicians were able to proceed. The newborn was admitted to NICU for respiratory distress and hypotonia and at examination he presented with upper limbs arthrogryposis, dorsally flexed feet, contractures at the knees and elbows, hypospadias and undescended testicles. He required feeding by nasogastric tube. A positive CMA confirmed Prader-Willi syndrome (paternal deletion) and pursuing testing for the familial history by ES identified a pathogenic variant in *COL6A2* in mother and son confirming a diagnosis of Ulrich congenital muscular dystrophy.

Case 69573001 was followed during pregnancy for a single umbilical artery and increased nuchal fold since the first trimester. Craniofacial features including macrocephaly with frontal bossing, hypertelorism, and micrognathia were observed in the second trimester. In the third trimester, large for gestational age fetal measurements were recorded, and polyhydramnios developed gradually that required fluid reduction and presented the opportunity for genetic testing refused previously. QF-PCR and CMA were reported as normal. The fetus was further assessed at birth and ES was pursued. She had Chiari I malformation and squamous synostosis that was followed conservatively. Results of ES confirmed the presence of two de novo disorders: Costello syndrome, and ERF-related craniosynostosis.

The presence of ultrasound abnormalities mirrored in postnatal findings in all cases that had ES positive results may have contributed to the impetus of the clinicians pursuing further diagnosis in these cases, as well as the decision of the parents to consent for further testing. In almost half of the cases with positive ES results there was a single system involvement identified prenatally, with additional features that complemented the clinical picture postnatally. Although more information was gained about the newborn postnatally (on craniofacial features, and systems whose prenatal assessment is limited including gastrointestinal, genitourinary), the prenatally available features would have been sufficient to obtain a diagnosis should ES have been available in an ongoing pregnancy setting (Table 2). Given our testing restrictions over the time the study was conducted, we are not able to comment how many patients would have opted for more testing, including ES, if that had been available prenatally.

Fetuses tested by NGS modalities overall showed as top systems affected to be craniofacial as seen previously [21], cardiovascular, and musculoskeletal systems (Figure 3). We are confirming that between panels and ES, the majority of the diagnoses obtained in our study were due to single gene changes and not copy number variants (41 vs. 33). These findings may help guide expectations and prioritization of tests based on body system, and the justification of implementing ES in ongoing pregnancies. Although the overall number of cases in which ES was completed are small, we underline the fact that the majority of these fetuses are living, and that may be a convincing element in deciding further testing for the patients in our cohort. Exploring parental choices in our population should be an important avenue for future studies.

As positive family history was one of the primary referral reasons for many of the cases evaluated, it is of particular importance to continue exploring potential diagnoses after a negative genetic test when other individuals in the family may contribute to the understanding of a less clear prenatal phenotype. Re-analysis is becoming more acceptable as a clinical workflow step in the modern genetic clinic as it enhances diagnosis over time, especially in relation to ES [22]. We suggest that reanalysis should become routine in the prenatal clinic for all the negative or unclear results regardless of testing modality as more data are shared worldwide and available databases are improved. We suggest that it is particularly important to revisit cases in PG in order to characterize clinical phenotypes at earlier stages as more diagnoses are achieved. An additional important tool is the use of matching websites such as Matchmaker exchange [23] and similar [24] that, again, over time may contribute to the delineation of new human conditions. Such a process is under investigation for one of our ES negative cases with a finding in a candidate gene not yet confirmed to be associated with a human phenotype (Table 2).

Our retrospective chart study has a number of limitations. It did not include results from a first trimester screen, QF-PCR, or karyotype, so these diagnoses were not captured in our review as the goal was to focus on more advanced means of diagnosis either through a genomic approach (CMA, ES) or targeted testing (panels). We have not captured details regarding the ethnic background of our cohort. Our study was limited by local logistics regarding the organization of the prenatal genetics clinic not incorporating the geneticists in the maternal Fetal Medicine care provider team over the timeline targeted by the study. In addition, pregnant women represent a population in which consistent follow up for prenatal testing may prove difficult for many. However, more than half of the patients assessed underwent testing. ES was available only postnatally and applied to a presumably restricted number of cases than if offered in ongoing pregnancies. Secondary findings were not recorded for this study or the modality of performing ES (single vs. duo vs. trio approach). It is not possible to know what the uptake of ES would have been if offered in ongoing pregnancies. However, by comparing to the introduction of CMA a few years back, once available, families have been increasingly open to explore the additional diagnostic tools that may bring light on their fetus’ presentation. This is also supported by other parent’s experience with the introduction of ES elsewhere [25]. An important limitation of genetic tests includes the identification of VUSs which adds complexity to interpretation of results, to counseling and do not offer further avenues in the clinical setting. As general understanding of the genetic basis of genetic disorders improves, previous negative or VUS results can be reexamined with a more critical eye not only regarding ES but also CMA and gene panels.

Our results show that with the strategy of genetic testing used during the period of this study, two thirds of the tested cases remained without a diagnosis. Our study has not included diagnoses reached by QF-PCR and karyotype, and this may have enhanced the overall testing yield observed. Based on current literature, it would be expected that by utilizing all the genetic testing modalities (QF-PCR, karyotype, CMA, panels, ES) up to two thirds of the pregnancies will receive a diagnosis [16]. While parental decision is paramount to performing testing, and a level of unpredictability regarding decisions in a highly emotionally charged clinical environment like PG remains, it is opportune to ask how the implementation of ES in ongoing pregnancies may change parental optics and uptake of the test overtime in our setting. A prospective study with ES employed in ongoing pregnancies may answer that question.

## 5. Conclusions

Improved access to prenatal testing in the form of CMA, gene panel testing, and ES has vastly expanded the possibilities of reaching prenatal diagnoses in a timely fashion. With the introduction of ES in ongoing pregnancies as a routine test, this diagnostic potential will be further enhanced. CMA and panel sequencing may continue to play an important role in prenatal diagnosis, the latter especially in well-characterized phenotypes (e.g., skeletal dysplasias), or when access to ES is lacking. Single anomalies should not be a deterrent for the use on ES in the prenatal setting. Embedding prenatal genetic professionals in the Maternal Fetal Medicine clinics, sustained access to thorough imaging fetal evaluations, and collection of detailed family histories remain quintessential in prenatal genetics of a contemporary prenatal care center.

Based on the results of this study, we conclude that access to genetic testing including ES in ongoing pregnancies in a public health care system will contribute directly to increasing the yield of prenatal diagnoses, empower families and health care providers involved in the difficult path of pregnancy decision making to achieve best patient-centered pregnancy management.

## Figures and Tables

**Table 1:** Prenatal assessed cases. Data of both prenatal and postnatal panel testing were achieved.

a. Prenatally Assessed Cases for Which Pre- and Postnatal Positive Results of Gene Panels Were Obtained *.									
Stud-y ID	Reason for Referral	Anomalies Identified		Prenatal Genetic Testing	Pregnancy Outcomes	Postnatal Genetic Testing	Diagnosis	Gene	Management Implications (RR%)
		Prenatal Detection	Postnatal Detection						
**Prenatal testing**									
67573	AbN US	9	9	(-) RAD, CMA (+) SD panel	TOP	nil	Hypochondrogenesis	*COL2A1*	<1%
66234	AbN US	9	9	(-) RAD, CMA (+) SD panel	Stillbirth	nil	Opsismodysplasia	*INPPL1*	<1%
66328	AbN US	3, 4, 5, 6, 7	3, 4, 5, 6, 7, 11	(-) RAD, CMA (+) Noonan panel	TOP	nil	Noonan syndrome	*PTPN11*	<1%
66330	AbN US	1, 5, 9	1, 5, 9	(-) RAD, CMA (+) Arthrogryposis panel	Nn demise	nil	Nemaline myopathy	*NEB*	25%
66883	AbN US	7, 8	3, 7, 8	(-) RAD, CMA (+) BW panel	Living	nil	Beckwith-Wiedemann syndrome	*KvDMR2* loss of methylation	<1%
66944	FHx	nil	11	(-) RAD, CMA (+) SCID panel	Living	nil	SCID	*IL2RG*	25%
67196	AbN US	3	1, 3, 10	(-) RAD, CMA (+) Noonan panel	Living	nil	Noonan syndrome (MIM163950)	*PTPN11*	<1%
67478	FHx		10	(-) CMA (+) Targeted panel	Living	nil	EBS (MIM131800)	*KRT14*	50%
67497	AbN US	9	9	(-) RAD, CMA (+) SD panel	Living	nil	Hypophosphatasia	*ALPL*	25%
67797	AbN US	1, 2, 6	1, 3, 5, 6, 7	(-) RAD, CMA (+) Noonan panel	Stillbirth	nil	Noonan syndrome	*PTPN11*	<1%
67981	AbN US	3, 4	3, 4	(-) RAD, CMA (+) Brain malformation panel	TOP	nil	*L1CAM*-related disorders	*L1CAM*	25%
68743	AbN US	7	7	(-) RAD, CMA (+) Congenital diarrhea panel	Living	nil	Congenital diarrhea	*SLC26A3*	25%
58884	AbN US	2, 5, 9	5, 9	(-) RAD, CMA (+) SD panel	stillbirth	nil	Thanatophoric dysplasia	*FGFR3*	<1%
**Postnatal testing**									
67259	AbN US	9	3, 9	nil	TOP	(-) CMA (+) SD panel	OI2	*COL2A1*	<1%
68120	AbN US	4, 8	4, 8	nil	TOP	(-) CMA (+) Ciliopathy Panel	Joubert Syndrome	*RPGRIP1L*	25%
68445	FHx	9	9	nil	TOP	(-) CMA (+) SD panel	Thanatophoric dysplasia	*FGFR3*	<1%
69205	AbN US	4, 9	3, 4, 8, 9	nil	Infantile demise	(-) CMA (+) Brain malformation panel	Zellweger syndrome	*PEX1*	25%
66125	AbN US	2, 6	1, 2, 3, 4, 6, 9	(-) RAD, CMA	Living	(+) Craniosynostosis panel	Acromelic fronto-nasal dysostosis	*ZSWIM6*	<1%
67274	AbN US	6, 7	1, 3, 4, 6, 7	(-) RAD, CMA	Nn demise	(+) Aicardi-Goutieres panel	Aicardi-Goutieres syndrome	*RNASEH2C*	25%
67502	AbN US	3	1, 3, 9	(-) RAD, CMA	Living	(+) Stickler syndrome panel	Stickler syndrome	*COL11A1*	50%
7009	FHx	nil		nil	Living	(+) MH panel	Malignant Hyperthermia	*RYR1*	50%
17890	FHx	nil	1, 3	nil	Living	(+) CDL panel	Cornelia de Lange syndrome	*NIPBL*	50%
63327	AbN US	3, 9	3, 9	nil	Nn demise	(+) Craniosynostosis panel	Beare-Stevenson syndrome	*FGFR2*	<1%
64860	FHx	nil	1, 3, 9	nil	Living	(+) OI panel	Bruck Syndrome	*FKBP10*	25%
66134	FHx	nil	11	nil	Living	(+) DSPD panel	Delta Storage pool disease	*RUNX1*	50%
66206	FHx	nil	1, 9	nil	Living	(+) Hposhosphatemia panel	Hypophosphatemia	*PHEX*	50%
66428	AbN US	6	3, 6	nil	Living	(+) Brain malfomation panel	Tuberous Sclerosis	*TSC1*	1%
67255	AbN US	9	1, 3, 9	nil	Nn demise	(+) SD panel	Campomelic dysplasia	*SOX9*	<1%
67739	AbN US	1, 3, 9	1, 3, 9	(-) RAD	TOP	(+) SD panel	Thanatophoric dysplasia	*FGFR3*	<1%
68014	FTS+	1, 2, 3, 5, 7	3, 5, 10	nil	Living	(+) Vascular malformation panel	*RASA1*-related disorder	*RASA1*	<1%
69624	FHx	3, 9	3	nil	Living	(+) Craniosynostosis panel	Saethre-Chotzen syndrome	*TWIST*	50%
67669	FHx	2, 9	3, 4, 9	nil	Living	(+) Congenital muscular dystrophy panel	Myotonic dystrophy type 1	*DMPK*	50%
**b. Prenatally assessed cases for which pre- and postnatal variants of unknown significance (VUS) results of gene panels were obtained.**									
59041	FTS+	1, 3, 4, 6, 9		(-) RAD, CMA (?) Panels (hydrops, brain malformations)	TOP	(?) WES	AR combined oxidative phosphorylation deficiency 27/Alpers-Huttenlocher syndrome	*CARS2* (NM_ 024537.4): c.563C>T,p.Thr188Met	25%?
67291	FTS+	3, 8	1, 3, 4, 9	(-) RAD, CMA (?) Noonan Panel	Living	nil	Noonan syndrome? Lost to follow up	*LZTR1* (NM_006767.3):c.2063G>A,p.(Arg688His)	?
67347	FHx	nil	nil	(-) RAD, CMA (?) Bleeding disorder panel	Living	nil	Bleeding disorder	*ABCG5*(NM_022436.2):c.235G>A,p.(Gly79Arg); *FREM1*(NM_144966.5):c.987A>T,p)Lys329Asn); *MPL*(NM_005373.2):c.655C>G,p(Gln219Glu)	?
67980	AbN US	6	6, 8, 11	(-) RAD, CMA (?) Noonan syndrome/Rasopathies	Living	nil	Complex syndromal presentation	*KAT6B*(NM_0123303.3): c.1217G>A, p.(Arg406Gln)mat	?
68077	AbN US	9	1, 9	(-) RAD, CMA (?) SD Panel	Living	nil	Pseudohypoparathyroidism	*GNAS*(NM_000516.4):c.349G>A,p.(Val117Met),mat	50%?
67042	AbN US	4, 6	4, 6	(-) RAD, CMA	Living	(-) CMA (?) Brain malformation panel	Joubert syndrome	*TUBA8*(NM_018943.2):c.1118G>A,p.(Arg373Gln), het *VLDLR*(NM003383.3):c.836G>A,p(Arg279Gln),het	?
66890	FHx	3, 9	3, 9	nil	TOP	(-) CMA (?) Arthrogryposis panel	Beals syndrome	*FBN2*(NM_001999.3):c.8363C>T,p.(Ala2788Val), het	?
67846	AbN US	2, 8	8	nil	Stillbirth	(-) CMA (?) CAKUT Panel	Bilateral Renal agenesis	*FREM2*(NM_207361.6):c.9038C>T,p.(Thr3013Met), het	?
68247	AbN US	3, 8, 9	3, 8, 9	(-) RAD	TOP	(-) CMA (?) Arthrogryposis panel	Rothmund-Thmpson syndrome	*RECQL4*(NM_004260.3): c.551G>A,p.(Gly184Asp), hom	25%?
66794	AbN US	1, 2		(-) RAD, CMA	Infantile demise	(?) Hydrops Panel	Feingold syndrome	*MYCN*(NM_005378.4):c.718G>A,p.(Gly240Ser)	?
66824	AbN US	1, 2, 9		nil	TOP	(-) CMA, (?) Hydrops Panel	Desbuquois dysplasia	*CANT1*(NM_138793.3):c.872C>T,p(Thr291Met), het	?

* Legend for corresponding anomalies: 1 = Growth Restriction or Syndromic (e.g., hydrops) anomaly, 2 = Abnormal Amniotic Fluid, 3 = Craniofacial or neck anomaly, 4 = Central Nervous system anomaly, 5 = Thoracic anomaly, 6 = Cardiovascular anomaly, 7 = Gastrointestinal anomaly, 8 = Genitourinary anomaly, 9 = Musculoskeletal anomaly, 10 = Skin and adnexa.

**Table 2:** Prenatal whole exome sequencing cases.

a. Prenatal Cases in Which a Diagnosis Was Achieved via Whole Exome Sequencing									
Study ID	Reason for Referral	Anomalies Identified		Prenatal Genetic Testing	Pregnancy Outcome	Postnatal Testing	Diagnosis	Genetic Finding	Management Implications
		Prenatal Detection	Postnatal Detection						
64702001	AbN US	3, 4, 6, 7, 8	3, 4, 6, 7, 8	nil	TOP	(-) RAD (-) CMA (+) WES	Developmental delay with or without dysmorphic facies and autism (MIM618454) AD	*TRRAP* (NM_003496.3), c.3127G>A, p.(Ala1043Thr), heterozygous, de novo	RR < 1%
71280001	AbN US	3, 4, 6	3, 4, 6, 7, 8	(-) RAD, CMA, Panel	TOP	(+) WES	Neurooculocardio-genitourinary syndrome (MIM618652), AD	*WDR37* (NM_014023.3), c.389C>T, p.(Thr130Ile), heterozygous, de novo	RR < 1%
71354001	AbN US	5, 6	3, 5, 6, 7, 8, 9	(-) RAD, CMA, Panel	Neonatal Demise	(+) WES	Osteopathia striata with cranial sclerosis (MIM 300373), XL	*AMER1* (NM_152424.4), c.19G>&, p.Glu7 *; hemizygous, maternal	RR25%
67893001	AbN US	6	6	(-) RAD, CMA in Alberta (+) WES in another province	Living	nil	1. RASA1-related disorder (MIM 608354), AD 2. Kleefstra sdr. type 2	1.*RASA1* (NM_002890.2), c.2131C>T,p.Arg711X; heterozygous, maternal 2.*KMT2C* (NM_170606.2), c.9773A>C, p.His3258Pro heterozygous, de novo	Surveillance 50% RR
66175001	AbN US	3	3, 9	(-) RAD, CMA	Living	(-) CLP Panel (+) WES	Hartsfield syndrome (MIM615465), AD	*FGFR1* (NM_023110.2), c.1474G>A,p.(Val492Met), heterozygous, de novo	Anticipatory management RR < 1%
67295001	AbN US	9	3, 9	nil	Living	(+) CMA (+) WES	1. Prader-Willi Syndrome (MIM176270), imprinting 2. Ullrich Muscular Dystrophy (MIM254090), AD	1.*Del 15q11*, paternal 2.*COL6A2*(NM_001849.3), c.848G > A,p.Gly283Glu, heterozygous, maternal	Treatment Anticipatory management RR 50%
69829001	AbN US	1, 4, 5, 9	1, 3, 4, 5, 9	nil	Living	(-) CMA (+) WES	Escobar Syndrome (MIM265000), AR	*CHRNG* (NM_005199.4), c.202C>T, p.Arg68 *; c.459dup, p.Val154Serfs*24, combined heterozygous, parents carriers	Anticipatory management RR25%
69573001	AbN US	1, 2, 3	1, 2, 3, 6	(-) RAD, CMA	Living	(+) WES	1. Costello Syndrome (MIM218040),AD 2. ERF-related disorder (MIM600775),AD	1.*HRAS* (NM_005343.3), c.34G>A, p.(Gly12Ser), heterozygous, de novo 2.*ERF* (NM_006494.3), c.891_892del, p.(Gly299Argfs * 9), heterozygous, de novo	Anticipatory management RR < 1%
68143001	AbN US	6	3, 6, 7	(-) RAD, CMA	Demise at 4 mo	(-) Panel (+) WES	Zhu-Tokita-Takenouchi-Kim syndrome (ZTTKS),AD	*SON* (NM_032195.2), c.5867G>A, p.(Arg1956His), heterozygous, de novo	RR < 1%
**b. Prenatal Cases in which WES identified a Variant of Unknown Significance**									
67663001	AbN US	3	3, 4	nil	Living	(+) CMA (-) CLP Panel (?) WES	1. 15q13 dup syndrome; 2. Intellectual Developmental disorder, auto-somal dominant 52	1. Paternal 2. *ASH1L* (NM_018489.2): c.614A>C,p.(Lys205Thr), pat	50%
68424001	FHx	2, 6, 7, 8	2, 3, 6, 7, 8, 9	nil	TOP	(-) RAD, (-) CMA (?) WES	CDX2 variant, gene not associated with human phenotype	*CDX* (NM_001265.4): c.592C>T,p.(Arg198Trp), de novo	<1%
67572001	AbN US	4, 9	3, 4, 9	nil	TOP	(-) CMA (?) WES	RYR3 variants, not associated with human phenotype	*RYR3* (NM_001036.4): c.90T>A,p.(His30Gln), pat C,11730G>C,p.Leu391Phe),mat	25%
69260001	AbN US	6, 7, 9	6, 7, 9	(-) RAD, CMA, Panel	TOP	(?) WES	Mandibulofacial dysostosis, Guyon Almeida type (MIM610536)	*EFTUD2*(NM_004247.3):c.658C>T,p.(Arg220Cys), de novo	<1%

* Legend for corresponding anomalies, 1 = Growth Restriction or Syndromic (e.g., hydrops) anomaly, 2 = Abnormal Amniotic Fluid, 3 = Craniofacial or neck anomaly, 4 = Central Nervous system anomaly, 5 = Thoracic anomaly, 6 = Cardiovascular anomaly, 7 = Gastrointestinal anomaly, 8 = Genitourinary anomaly, 9 = Musculoskeletal anomaly.

## Data Availability

Not applicable.

## Supplementary Materials

The following supporting information can be downloaded at: https://www.mdpi.com/article/10.3390/genes13112019/s1. Figure S1: Summary of test results for cases who had CMA prenatal, CMA postnatal, Panels prenatal, Panels postnatal, and WES postnatal; Figure S2: Distribution of fetal features by systems in relation to positive NGS-based tests (prenatal and postnatal gene panels and postnatal WES); Figure S3: Clinical workflow for testing for patients who had prenatal CMA; Figure S4. Clinical workflow for patients who had CMA either postnatally or both prenatally and postnatally; Figure S5. Clinical workflow for patient who did not have CMA or had it postnatally

## Abbreviations

CMA	Chromosomal microarray
CNS	Central nervous system
CV	Cardiovascular
ES	Exome sequencing
FHx	Positive family history
FTS	First trimester screen
GA	Gestational age
GI	Gastrointestinal
MRI	Magnetic resonance imaging
MSK	*Musculoskeletal*
NGS	Next generation sequencing
PP	Previous pregnancy
PC	Previous child
QF-PCR	Quantitative Fluorescent*-Polymerase chain reaction*
WES	Whole exome sequencing
VUS	Variants of unknown significance
?	Variants of unknown significance
