# Supplementary material for: Prenatal Genetic Testing in the Era of Next Generation Sequencing: A One-Center Canadian Experience
Source: Genes (Basel). 2022 Nov 3;13(11):2019. doi: 10.3390/genes13112019 (PMC9690880; doi:10.3390/genes13112019)
Supplement: Supplementary file 1 [file genes-13-02019-s001.zip › genes-1975769-supplementary.pdf]

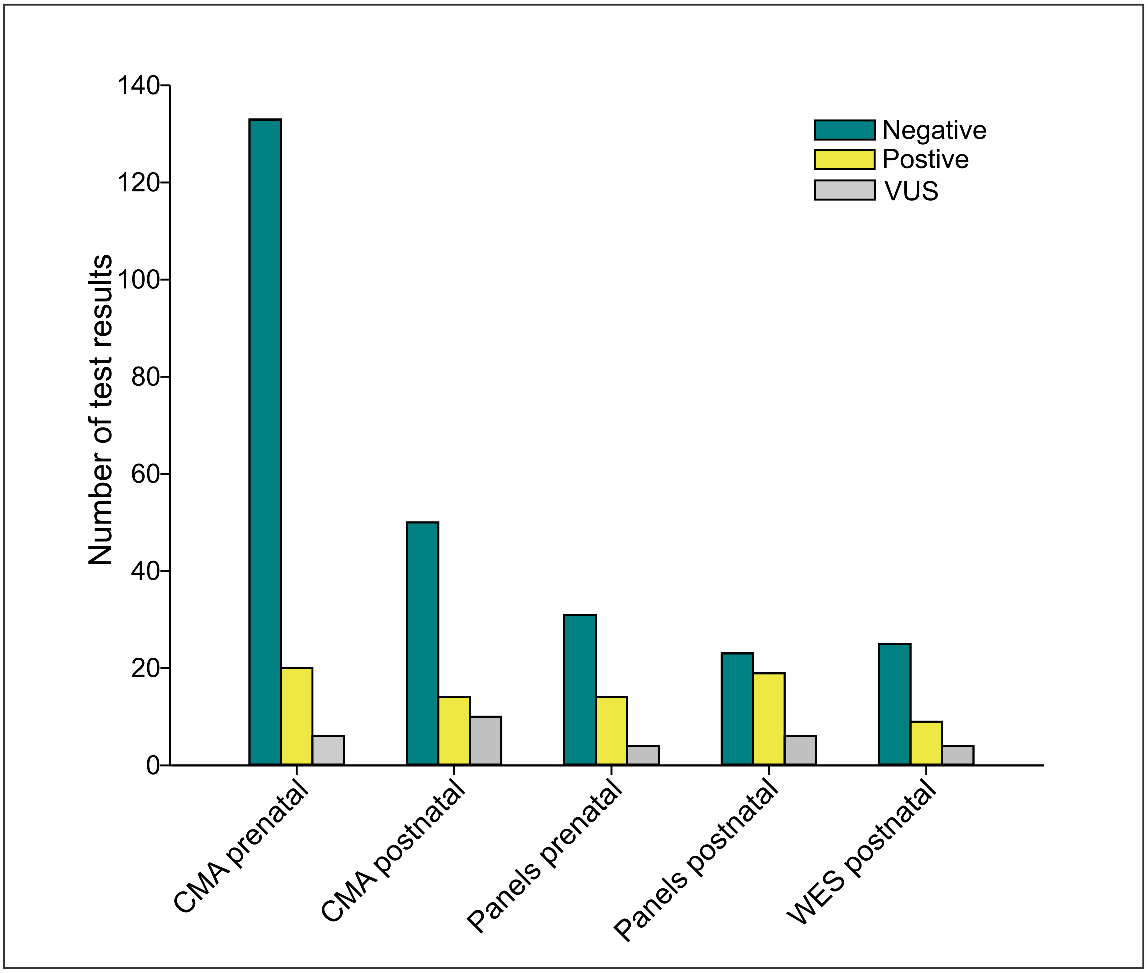

Figure S1. Summary of test results for cases who had CMA prenatal tests, CMA postnatal, Panel prenatal, Panel postnatal, and WES postnatal tests).

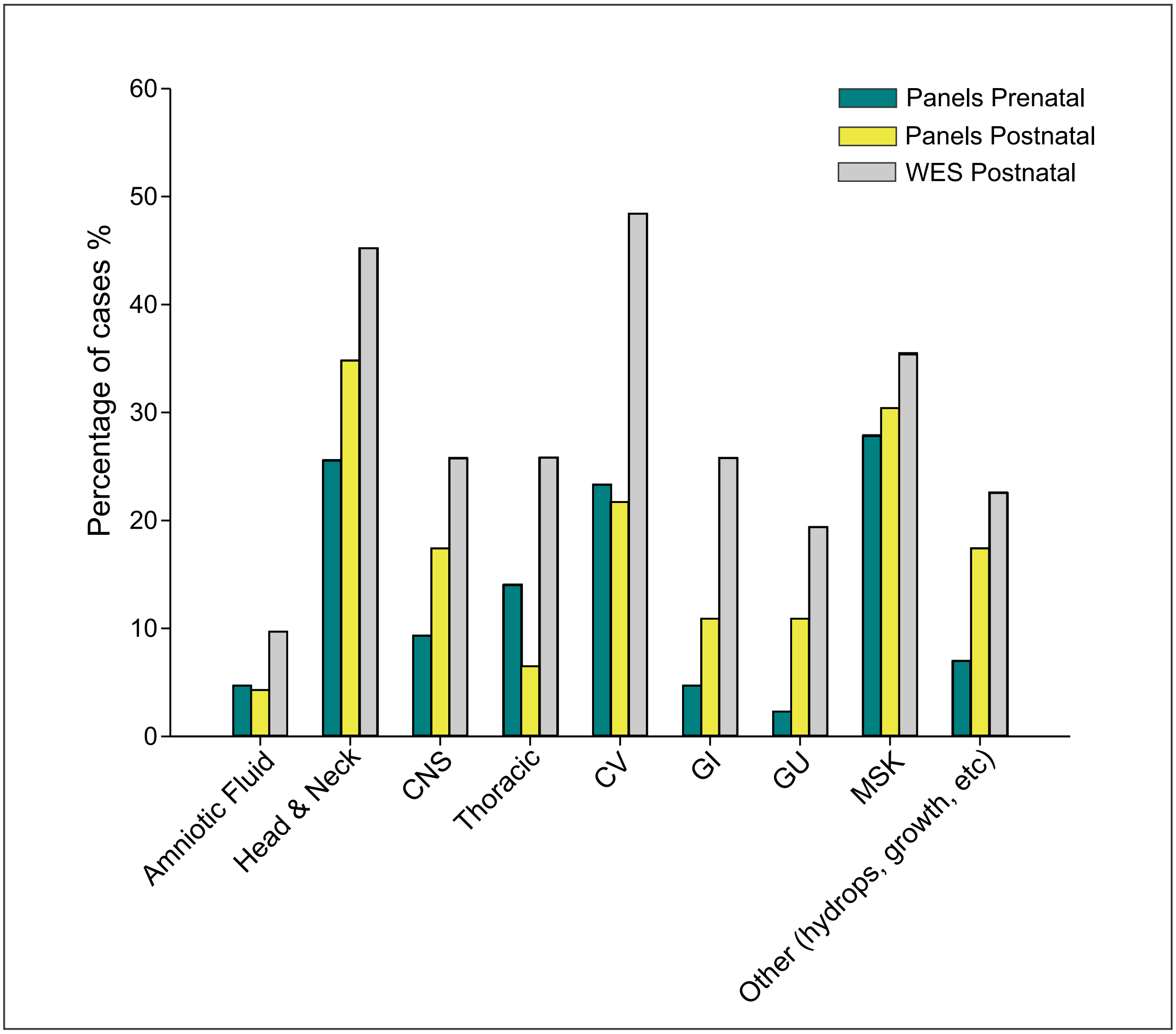

Figure S2. Distribution of fetal features by systems in relation to positive NGS-based tests (prenatal and postnatal gene panels and postnatal WES).

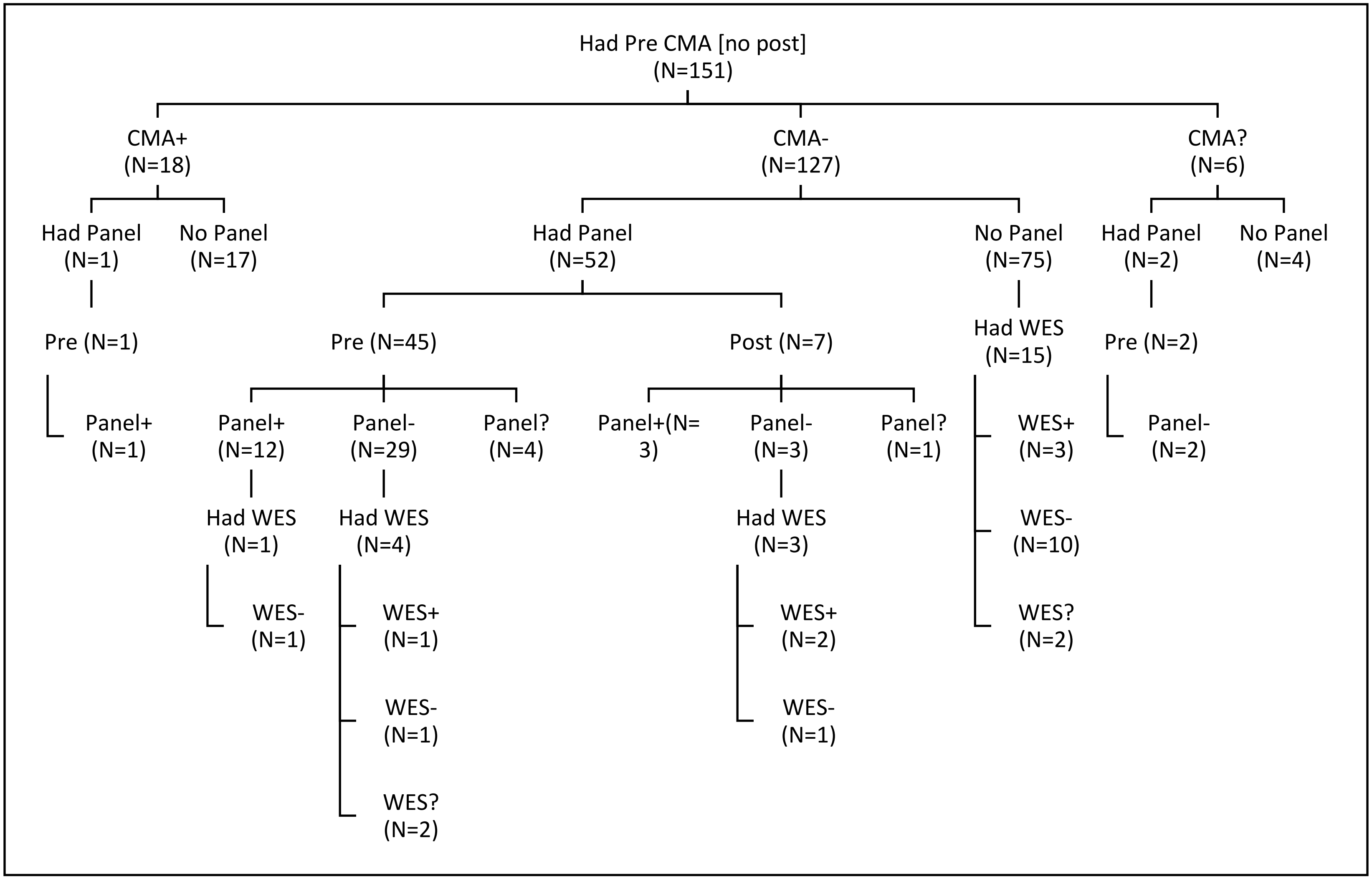

Figure S3. Clinical workflow for testing for patients who had prenatal CMA testing

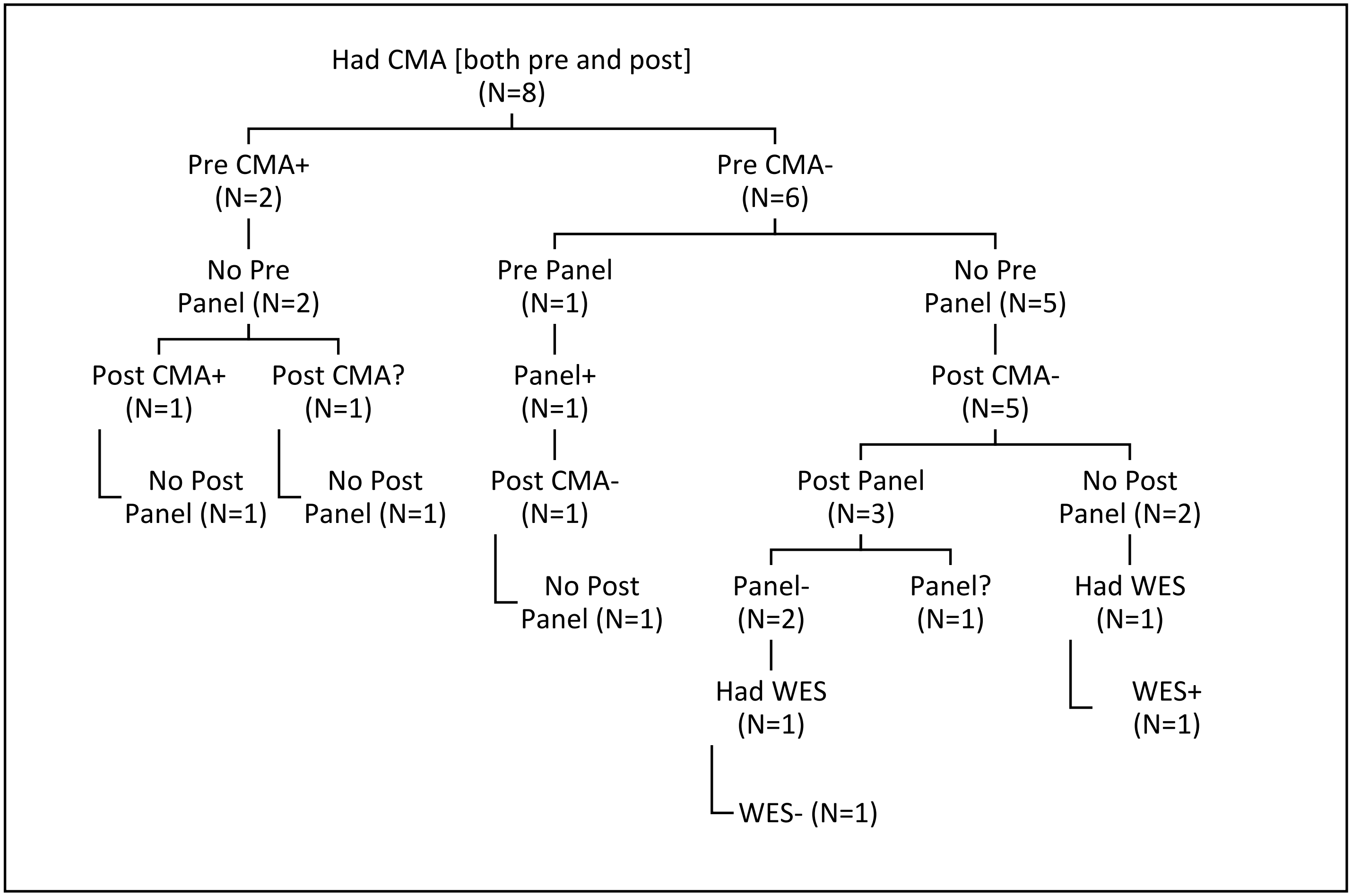

Figure S4. Clinical workflow for patients who had CMA either postnatally or both prenatally and postnatally.

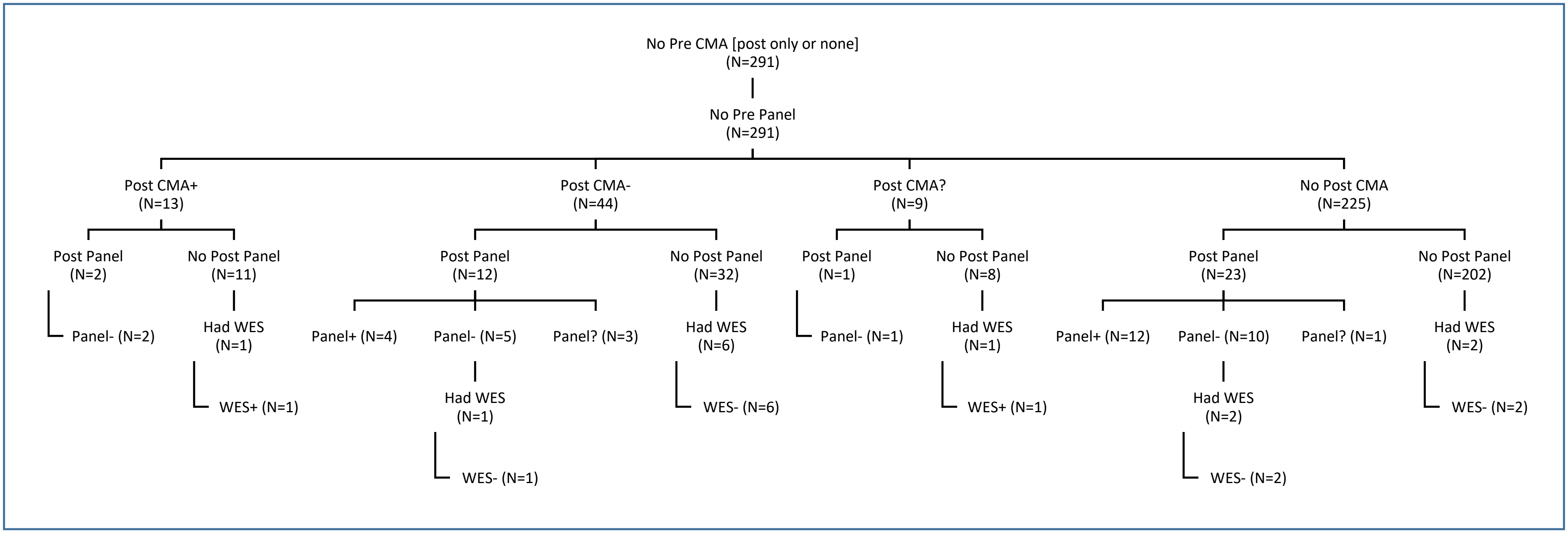

Figure S5. Clinical workflow for patient who did not have CMA or had it postnatally.
